# Hospital Website Rankings in the United States: Expanding Benchmarks and Standards for Effective Consumer Engagement

**DOI:** 10.2196/jmir.3054

**Published:** 2014-02-25

**Authors:** Timothy R Huerta, Jennifer L Hefner, Eric W Ford, Ann Scheck McAlearney, Nir Menachemi

**Affiliations:** ^1^Departments of Family Medicine and Biomedical InformaticsCollege of Medicine, The Ohio State UniversityColumbus, OHUnited States; ^2^Department of Family MedicineCollege of Medicine, The Ohio State UniversityColumbus, OHUnited States; ^3^Bryan School of Business and EconomicsUniversity of North Carolina GreensboroGreensboro, NCUnited States; ^4^Department of Health Care Organization and PolicyUniversity of Alabama at BirminghamBirmingham, ALUnited States

**Keywords:** social media, hospitals, information services, communication, access, consumer health information

## Abstract

**Background:**

Passage of the Patient Protection and Affordable Care Act (ACA) increased the roles hospitals and health systems play in care delivery and led to a wave of consolidation of medical groups and hospitals. As such, the traditional patient interaction with an independent medical provider is becoming far less common, replaced by frequent interactions with integrated medical groups and health systems. It is thus increasingly important for these organizations to have an effective social media presence. Moreover, in the age of the informed consumer, patients desire a readily accessible, electronic interface to initiate contact, making a well-designed website and social media strategy critical features of the modern health care organization.

**Objective:**

The purpose of this study was to assess the Web presence of hospitals and their health systems on five dimensions: accessibility, content, marketing, technology, and usability. In addition, an overall ranking was calculated to identify the top 100 hospital and health system websites.

**Methods:**

A total of 2407 unique Web domains covering 2785 hospital facilities or their parent organizations were identified and matched against the 2009 American Hospital Association (AHA) Annual Survey. This is a four-fold improvement in prior research and represents what the authors believe to be a census assessment of the online presence of US hospitals and their health systems. Each of the five dimensions was investigated with an automated content analysis using a suite of tools. Scores on the dimensions are reported on a range from 0 to 10, with a higher score on any given dimension representing better comparative performance. Rankings on each dimension and an average ranking are provided for the top 100 hospitals.

**Results:**

The mean score on the usability dimension, meant to rate overall website quality, was 5.16 (SD 1.43), with the highest score of 8 shared by only 5 hospitals. Mean scores on other dimensions were between 4.43 (SD 2.19) and 6.49 (SD 0.96). Based on these scores, rank order calculations for the top 100 websites are presented. Additionally, a link to raw data, including AHA ID, is provided to enable researchers and practitioners the ability to further explore relationships to other dynamics in health care.

**Conclusions:**

This census assessment of US hospitals and their health systems provides a clear indication of the state of the sector. While stakeholder engagement is core to most discussions of the role that hospitals must play in relation to communities, management of an online presence has not been recognized as a core competency fundamental to care delivery. Yet, social media management and network engagement are skills that exist at the confluence of marketing and technical prowess. This paper presents performance guidelines evaluated against best-demonstrated practice or independent standards to facilitate improvement of the sector’s use of websites and social media.

##  Introduction

More than 80% of adults reported using Internet resources to support health care decisions in 2011 [1,2]. Thus, in many instances, a hospital’s home page is the first point of contact for consumers [3,4]. As a result, the website for a hospital or health system has become an important communication channel for marketing to current and potential customers, as well as to visitors accompanying a patient [5-7]. Customers’ evaluations of a hospital’s website, and by extension their perceptions of the facility itself, will be based in part on comparisons to their experiences using other consumer websites such as Amazon and eBay [8,9]. If a hospital’s website does not conform to or exceed a customer’s expectations based on their previous experiences, negative inferences about facility quality will influence the decision-making process [10].

Reliance on search engines has resulted in individuals taking varied routes to their website destinations. Weaver et al [11] found that people seeking illness-related information behave differently than those seeking wellness information. Based on these trends, many hospital or health system websites have begun to include tools and information for patients and visitors that make navigating complex health encounters more user-friendly and that create a positive organizational image [12]. In so doing, hospitals are increasingly seeking to take on the role of trusted adviser, a role that is closely aligned with the accountable care organization (ACO) model in which health care providers work to empower patients to improve population health [7,13,14].

The competitive advantage gained from building an effective Web presence has led researchers to establish accessibility, content, marketing, and technical standards that define best demonstrated practices in website design [15-17]. In addition, the Health Information Technology Institute has issued standards for health care websites, including credibility, content, disclosure, links, design, interactivity, and caveats [18]. As a result, an exploration of US hospital and affiliated health system websites against the design standards used in other commercial endeavors is warranted to establish the state of the field.

The purpose of this article is to identify the degree to which hospitals and their health system websites comply with Internet-industry standards for commercial usability [19]. Using an automated Web crawler, we evaluated four dimensions—accessibility, content, marketing, and technology—using weighted multi-item scales. In addition, we used a weighted composite overall score to measure each website’s quality across all four dimensions. The authors believe this analysis to be a census assessment for the online presence of hospitals and their health systems in the United States.

For hospital and health system decision makers, these analyses provide quantifiably objective, and immediately actionable, recommendations for enhancing the quality of their organization’s website. Compared to other health information technology (HIT) upgrades that are being made to meet meaningful use goals set by the federal government, the capital investments required to create a state-of-the-art website are relatively modest and immediately visible. Additionally, as outlined above, having an effective website can create a competitive advantage when attracting consumers.

There is a dearth of evaluation studies on hospital websites. A number of factors play into this issue. Much of the research on the role of the Web in health include issues of accuracy, understandability, provenance, and timeliness. Williams et al [20] discuss a typology offered by Trochim on the purpose of websites and note that while the organizations don’t keep their purposes neatly within the lines, they can still be assessed on the *raison d’être*—their reason for being and the services they aspire to provide. As such, there have been some efforts to study the content of health sites on a more specific basis—by judging the quality of the information as opposed to the technical merits of the system that is used for conveying that material. The American Public Health Association (APHA) offers a comprehensive statement on these issues [21].

First, while numerous studies exist focused on health information, hospitals are generally not seen as a portal for that purpose. This can be seen in the samples from which many of the health assessments are drawn—generally sites like WebMD, which seek to provide health information [20]—as opposed to local hospitals. While some facilities are large enough to have a national draw, and therefore serve the former purpose (eg, the Mayo Clinic, the Cleveland Clinic), most hospitals do not seek to provide health information, per se. Rather, they seek to provide information on services provided at the facility. As a result, hospitals serve a different role in the community, which generally is the delivery of health care services. This relationship is transforming as a result of the move to the ACO model in the United States, where hospitals are taking a greater responsibility for the care of the panels of patients they serve.

Ivory [22] notes that website evaluation is a moving target, suggesting that early assessments of evaluation focused on technical assessments of speed, while those have shifted over time. While some scholars have noted that human participation is fundamental to evaluating the usability of Web content, to ignore the value of automated assessment is to ignore the variability of experience provided to the end user by virtue of a site’s failure to adhere to standards. Chiang and Starren [23] detail an assessment of consumer health website accessibility by users with sensory and physical disabilities, noting that W3C compliance is a minimum standard for ensuring accessibility, and adherence to that standard protocol should be framed as foundational and a precursor to more detailed assessment.

## Methods

### Overview

The names, cities, and states for every “non-government, not-for-profit (NFP)” or “investor-owned, for-profit” general medical or surgical hospital listed in the AHA 2009 Annual Survey in the United States (N=3523) were used in both Google and Bing to identify the first three returns for the search engines. These links were inspected to identify whether a hospital matching the facility of interest could be identified. In cases where a matching facility could not be found, an additional manual search was conducted in Google to attempt to locate the facility. Web site domains were included if and only if they could be attributed to the facility or a parent organization that was also a health services delivery organization. For instance, several critical access hospitals were excluded because they maintained a Web presence under a county government’s Web domain. Website testing took place during the month of May 2013.

It should be noted that some organizations may have been a member of a health system, but also maintained their own domain. For instance, Northeast Arkansas Baptist Health System (neabaptist.com) is a hospital associated with Baptist Health System (baptistonline.org). In cases where a facility had its own domain, we assessed that domain separate and apart from the network or health system of which it was a part. The website of each organization was secured using a custom-built Web crawler. The Web crawler begins at the top-level Web page for the domain of each facility or system (eg, for the Kaiser Permanente domain, the Web crawler starts at the home page, kaiserpermanente.org), and drills down into successive subpages to build a topographical map of the links within a site. The analytic engine then samples 500 of these subpages and evaluates them based on a battery of assessment items, discussed at length in subsequent sections. A few websites were not assessed by the Web crawler due to technical problems, including timeout due to slow webpages or Web servers, server-side page redirections, or missing or unavailable host names. Only pages residing within the identified facility domain were tested.

To create summarized scores of website performance, the analytic engine scored content along five dimensions: (1) accessibility, (2) content, (3) marketing, (4) technology, and (5) usability. The scores on each dimension are reported on a range from 0 to 10, with a higher score on any given scale representing better comparative performance. The five dimensions detailed in the following sections provide broad assessments of aspects of website quality based on a set of underlying individual metrics. While it is important to note that some specific metrics contribute to more than one of the summarized scales, the scores themselves provide a basis for comparing two or more sites. The definitions of the specific items measured and how they are weighted in the summarized scores are presented in Multimedia Appendix 1.

### Accessibility Dimension

Accessibility is a critical factor for reaching as many users as possible, but at-risk groups may not be familiar with access features that require higher levels of computer literacy, such as hovering over highlighted phrases to see additional information. Given the service domain in health care, the issue of accessibility is all the more important, and much discussion has been held on issues of access to services [24,25]. The accessibility score is an assessment of a website’s ease of use for individuals with lower computer literacy levels, including those with physical disabilities that limit their use of a mouse or non-standard browser (such as mobile phones or tablet devices). A number of accessibility evaluation tools are offered by the World Wide Web Consortium (W3C) to explore these issues.

### Content Dimension

The content dimension is an assessment of a website’s overall content quality without taking into consideration the technical limitations of the site. Content quality is considered high if the text is grammatically correct, relevant, and updated regularly. The quality of the site’s imagery (ie, photos and graphics) and metadata (ie, information about the data content in specific locations) is also assessed. Elements contributing to the content scale include individual tests of spelling, the degree to which the site adds new material, and the calculated reading age of the text on the pages. In particular, the Flesch-Kincaid readability metrics used in other health-related website studies are included as part of the content analysis [24,25]. The major measures that contribute to the content scale are freshness and the amount of content. The freshness measure is calculated by reading the dates that appear on a website’s pages. Up-to-date content is a positive indicator to consumers that the organization is engaged in state-of-the-art activities. For example, monthly updates to the CEO’s message may be understood to imply that a facility is customer-focused, while out-of-date content may foster a perception that public impressions are less important to the organization. Therefore, routinely adding and changing content to remain current and explicitly documenting the dates that Web pages are updated should be standard practice.

### Marketing Dimension

The marketing dimension is an assessment of how readily and reliably information is accessed using search engines, including the appropriateness of content to hyperlinks, the rank and popularity of the website, and other technical aspects related to search engine optimization (SEO). SEO is an important aspect of the marketing scale. As content within a page becomes more accessible to search engines, the organization’s profile in online searches becomes higher. Contributing individual tests include search engine results, search placement, and the use of content keywords that search engines rely on to prioritize websites. Performing these tasks effectively helps health systems maintain a consistent corporate image [26].

### Technology Dimension

The technology dimension is an assessment of how well a website is designed, built, and maintained. Technical issues affect the user’s experience and therefore can have a direct impact on the overall utility of the website for making decisions. Elements contributing to the technology index’s scores include website download speed, site structure, code quality, and the use of cascading style sheets to organize content. The technology scale focuses purely on the performance aspects of a website without respect to its content. The major contributor to the score is the speed measure.

### Usability Dimension

The usability score is a cross-sectional composite of a number of metrics used in other scales; therefore, it is a composite of metrics, not a composite of the other four scales. This dimension attempts to answer the question of how good a particular website is. Having this at-a-glance metric that rates the overall quality of a website as a single number enables comparisons across a number of critical areas of site presentation. The analytic engine also provides clear information about how each individual organization performs and, by extension, offers clues as to how improvements in these scores might be made.

### Average Ranking Across Dimensions

Rank order calculations for each of the five dimensions were averaged to create a single average rank. The average rank score was then calculated across all website domains in order to rank hospitals from 1 to 2407.

## Results

This search produced websites associated with 2407 unique domain names. Of those Web domains, 378 were attached to multiple AHA identifiers, indicating that they were part of larger organizations (ie, members in a system). In these cases, the system’s domain was tested once rather than isolating a single facility from its parent organization. In total, 2785 facilities were scored. Organizational characteristics of these facilities are presented in Table 1.

**Figure 1:**
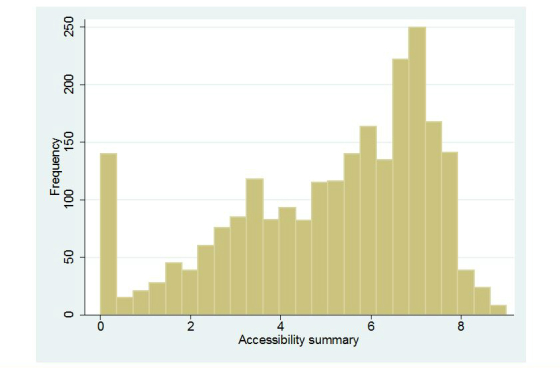
Distribution of scores: Accessibility.

**Figure 2:**
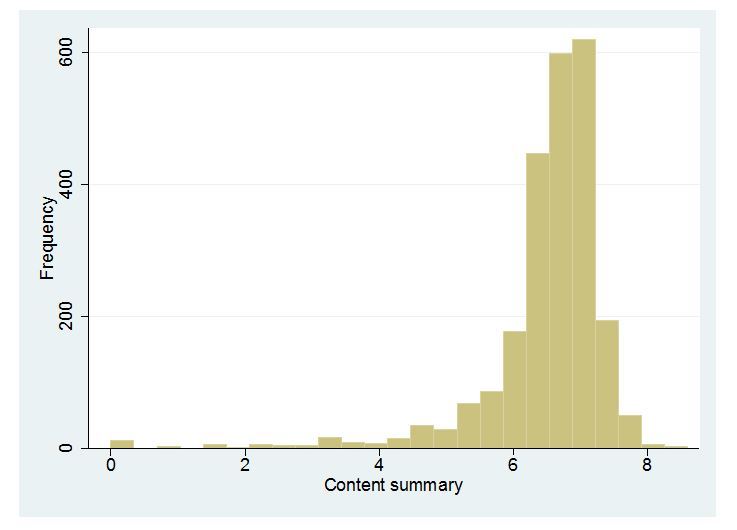
Distribution of scores: Content.

**Figure 3:**
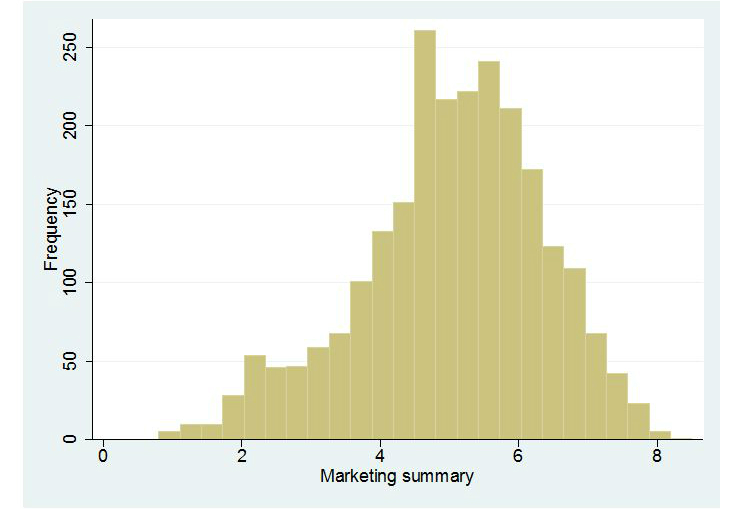
Distribution of scores: Marketing.

**Figure 4:**
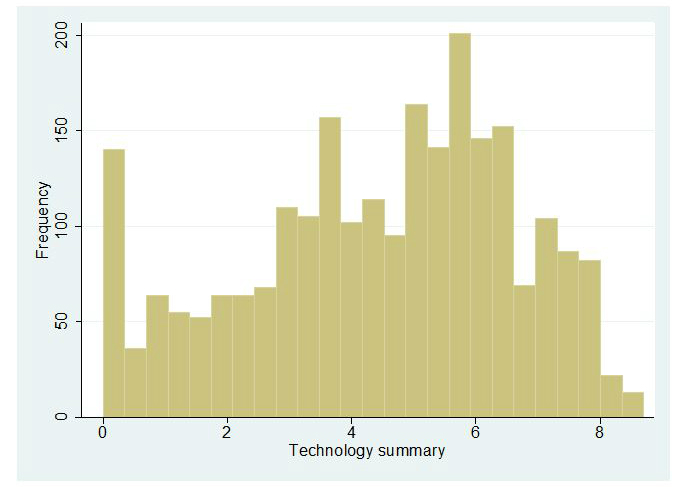
Distribution of scores: Technology.

**Figure 5:**
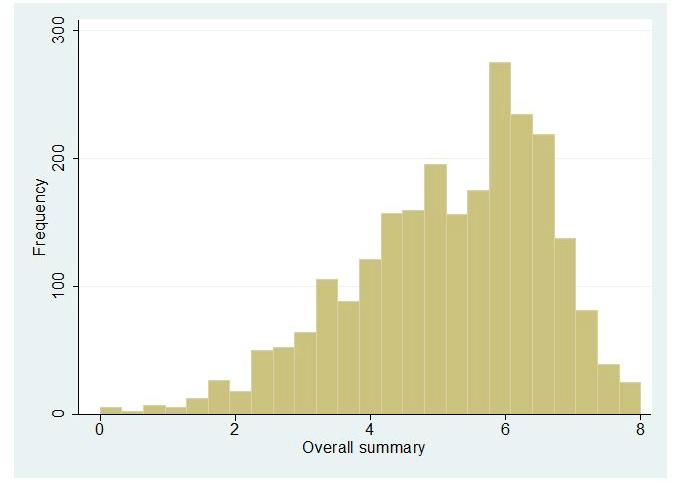
Distribution of scores: Overall.

Histograms representing the distribution of observations in each of the summarized scores are presented in Figures 1-5. Across all Figures, we find the scores to have a single mode with skewed distributions. The greatest variance can be seen in Figure 4, Technology. The mean scores for each dimension are presented in Table 2. Looking at the usability scale, the mean score was 5.16 (SD 1.43), with a maximum score of 8 achieved by only five organizations. Mean scores on the other scales ranged between 4.43 (SD 2.19) for technology and 6.49 (SD 0.96) for content.

The top 100 websites on each dimension are presented in Multimedia Appendix 2 with their respective rankings. The last column is an average rank score calculation across the five scales. For instance, jaxhealth.com scored 15^th^ in Accessibility, 61^st^ in Content, 52^nd^ in Marketing, 14^st^ in Technology, and 6^th^ in Usability—resulting in an average score of 29.6, and making it the 1^st^ best site overall. These rank order calculations, along with summary scores across dimensions, are presented for all 2407 websites in Multimedia Appendix 3. Included in these raw data is the AHA ID for each website domain.

**Table 1:** Hospital characteristics for all US AHA^a^ hospitals by inclusion in the study.^b^

Hospital characteristics	Matched	Not-matched	Total US AHA^b^ hospitals
Count of AHA IDs	2785.00	738.00	3523.00
Number of births	899.73	1035.24	928.12
Adjusted patient days	79,030.26	82,914.00	79,843.82
Transfer-adjusted admissions	15,299.73	16,595.29	15,571.13
Total expenditures	152,355,799.64	160,273,331.85	154,014,368.69
FTE employees	1011.65	1088.71	1027.80
Number of surgical operations	6387.38	7297.25	6577.98
Total visits	146,552.48	149,520.98	147,174.32
Number of beds	181.04	191.52	183.24
Average daily census	116.40	125.38	118.28

^a^AHA: American Hospital Association.

^b^Not-Matched Hospitals were excluded from the study because they did not have an identifiable Web domain attached to their name.

**Table 2:** Summary statistics for scales (n=2407).

Variable	Mean (SD)	Min	Max
Accessibility	5.08 (2.22)	0.0	9.0
Content	6.49 (0.96)	0.0	8.6
Marketing	5.03 (1.33)	0.8	8.5
Technology	4.43 (2.19)	0.0	8.7
Usability	5.16 (1.43)	0.0	8.0

## Discussion

### Principal Results and Implications

Those with an interest in evidence-based management know that one limitation is the presence of data upon which to make management decisions. While a website might have engaging photos and an interesting design, the proper functioning of a website requires far more work “under the hood” than most consider [27]. Layer upon that the importance of a broader social media strategy, and it can be easy to see how a facility might judge their online presence by the attractiveness of the pages. However, health care bears a special burden. With populations that are seeking care when they are least able to deal with externalities, a well-designed site that adheres to national standards and demonstrated best practices is important for issues related to care access [28,29]. Further, the absence of a comprehensive assessment across the entire sector could pose a significant detriment to care, given assumptions that may be made about accessibility and other factors. A thorough assessment requires the auditing of websites beyond the first pages. The present project, systematically assessing the website quality of 2785 hospitals, is a four-fold improvement over prior research and represents what the authors believe to be a census assessment for the online presence of US hospitals and their health systems.

The low mean score we found on the usability scale (5.16 on a 10-point scale, SD 1.43), a measure of general website quality, indicates that organizations’ websites, on average, have significant potential for improvement. In order to make a complete and effective assessment of a health care organization’s website, it is necessary to have the site evaluated using a Web crawling and analytic engine similar to the one employed in the present study. Nevertheless, there are many contributing scale components that organizational leaders can assess by a simple visual inspection of their website (eg, see the 2012 paper by Ford et al [30] for a more comprehensive discussion of this issue). Multimedia Appendix 1 presents a list of scale components with accompanying definitions and the percentage weight of each component within the five scales. These component definitions provide guidance for leaders to identify “low-hanging fruit” to improve their website scores. For example, one could easily search for and repair “Broken Links” (links to Web addresses that do not exist or return an error). Additionally, there is a “Spelling” component that assesses whether the words on a page are spelled correctly, and a “Twitter” component that determines if the website is linked to a Twitter account and how often tweets are posted.

While we did not test for the relationship between organizational type and website performance, a cursory review of the top 100 websites reveals that large facilities performed well. Consumers in urban areas typically go to a local facility for routine care or common procedures, but customers in rural areas may bypass their local facilities. In particular, consumers may travel for specialty care that is inaccessible locally, shopping for a facility and turning to the Internet for information [31,32]. Another explanation for large organizations’ success is that the complex and high-risk nature of cancer and childhood illnesses makes selecting an organization with high-quality ratings and a reputation for innovation particularly important [33,34]. This may contribute to the impetus for specialty facilities to develop high-performing websites for their marketing purposes.

For policymakers, the present analyses of health systems’ websites may provide an indication of whether or not a health system is striving to become an ACO. Given the scope of organizational change required to become an ACO, it stands to reason that a health system’s website would document and reflect such efforts in order to take advantage of that effort in the market. As a result, website quality may be a simple and reliable leading indicator of efforts to make this critical organizational transition. Prior research has found these dimensions of website quality to be linked to important aspects of patient care, including patient safety metrics [35], thus confirming the potential importance of our findings.

### Limitations

The authors note several limitations to this study. Facilities associated with an education top domain (.edu) were purposely excluded from the analysis. The decision to exclude the .edu-based hospitals was a difficult one. While these hospitals represent a significant type of player in hospital health care delivery, they also often contain an academic side that would skew assessment. It was therefore impossible to create decision rules to exclude pages that were not directly tied to patient care or the hospital. As a result, inclusion of .edu-based content might result in academic departments unrelated to care influencing measures. It should also be noted that in some instances, hospitals create a facility domain that is separate and apart from the academic center. In these cases, where the domain is .org or .com, we assessed that content, but excluded .edu content if it was linked on any page. Therefore, a facility might have their clinic included, but the information about their doctors residing on the academic side might be excluded. The study authors intend to assess this subgroup in future analyses.

A similar dynamic occurred, at times, with select websites purchased outside of the .com and .org domains. For instance, facilities have also registered on the “.info” and “.us” domains. In these cases, the authors attempted to determine if the identified site was owned by the facility. These were judgment calls on the part of the research team, potentially resulting in counting errors, which would manifest in the descriptive statistics.

Another limitation of the research is related to the emerging nature of health care facilities. With the contraction and centralization of health care, there are fewer independent hospitals. Increasingly hospitals are joining networks and systems of care. These dynamics are becoming more pronounced in the wake of ACO development efforts. As a result, we can increasingly expect that access to regional information will be moved further down into Web pages as these systems centralize their marketing and information dissemination functions. Additionally, it should be noted that many facilities keep their content in a secure environment for users of their health systems. As a result, the assessment might incorrectly assess their Web presence. The assessment only reaches what is not secured based on links accessible from the home pages. This would simulate the information available to either non-members or members using only publically available information.

Finally, the authors recognize a concern around size and scope and the potential to misrepresent a single score as sufficient explanation of information given the diverse nature of facilities. In some cases, a website for a facility can be 6 pages. In another case, the system’s website is over 10,000 pages and centrally managed. To then say that a domain is scored at 5.4 overall for both does not, in fact, mean they are equivalent. There are tradeoffs that any single measure must make. As a result, we have chosen to publish not only the overall score, but also the other tailored scores. The result is a greater nuance, but moves away from that single measure.

### Comparison With Prior Work

In 2011, the websites of 636 US hospitals and health systems were tested using a similar methodology, with an overall mean score of 6.37 [30]. While we acknowledge that this prior work displayed higher scores, the addition of so many new facilities makes comparisons to prior evaluations problematic. The present study assessed 2407 domains that covered 2785 facilities. This is a four-fold improvement in prior research and represents what the authors believe to be a census assessment for the online presence of US hospitals and their health systems. We expect smaller facilities to have lower scores, on average. Put another way, we expect a lowering of scores, in comparison to scores calculated from prior research, as a result of greater inclusivity in our current assessment. Future iterations of this study will permit greater comparability across years.

### Conclusions

The current analysis presents a significant update to the systematic assessment of hospital social media presence. Given the movement toward having health systems serve as ACOs that can empower consumers [14,30], the number of poorly performing facilities across all the calculated scores is concerning in the near term. The social media and Web presence of many of these organizations represents the first contact health care consumers make with the organization. If such contact fails to make a positive impression on the consumer, alternatives may be explored. In saturated markets where several organizations’ services are interchangeable, a strong and well-designed Web and social media presence can be the difference between patients taking the first step into a facility or doing everything they can to avoid it. Health organizations should strive to standardize the quality of information presented on their websites [36], but they should also take care to deal with issues of accessibility, standards compliance, and search engine optimization.

## Multimedia Appendix 1

Scale components and weightings.

## Multimedia Appendix 2

Ranking of the Top 100 websites for each dimension and an average ranking across dimensions.

## Multimedia Appendix 3

Complete dataset including domains, associated composite scores, and rankings. (Crosswalk data provided in separate sheet needs to be merged to use the dataset. Not provided merged due to a concern that univariate analysis of the merged data will multiply count single observations, because facilities may share a domain).

## Abbreviations

ACO: accountable care organization
AHA: American Hospital Association
SEO: search engine optimization
