# Supplementary material for: Hospital Website Rankings in the United States: Expanding Benchmarks and Standards for Effective Consumer Engagement
Source: J Med Internet Res. 2014 Feb 25;16(2):e64. doi: 10.2196/jmir.3054 (PMC3961706; doi:10.2196/jmir.3054)
Supplement: Supplementary file 1 [file jmir_v16i2e64_app1.pdf]

**Multimedia Appendix 1**
**Scale components and weightings**

| Assessment components | Description | Accessibility | Content | Marketing | Technical | General | Usage across all categories |
|---|---|---|---|---|---|---|---|
| Alternative text | Assesses whether alternative text accompanies images | 15% | | 4% | 4% | 5% | 6% |
| Amount of content | Measures the number of pages with a reasonable amount of text | 8% | 33% | 9% | | 5% | 9% |
| Analytics | Determines whether website implements software packages to track new and recurring visitors, which web pages readers visit and for how long, etc. | | | 4% | 4% | 3% | 3% |
| Broken links | Assesses whether the site contains links to web addresses that do not exist or return an error | 8% | | 1% | 8% | 5% | 4% |
| Domain age | Identifies the original registration date of the organization's domain to determine age. This is a component of SEO systems | | | 4% | | | 1% |
| Error pages | Checks for error messages and faults. Such errors are strongly suggestive of technical problems in the website | | | 4% | 8% | 3% | 3% |
| Facebook | Checks whether the website has a Facebook page, Facebook group, or personal Facebook profile and scores that site by the number of likes published by Facebook | | | 8% | | 4% | 3% |
| Headings | Assesses whether headings are used effectively throughout the site to improve search engine placement, accessibility, and usability | 8% | | 4% | 8% | 5% | 5% |

| | | | | | | | |
|---|---|---|---|---|---|---|---|
| Incoming links | Measures the number of links from major search engines, including Yahoo! and Google | | | 9% | | 5% | 4% |
| Link states | Assesses the use of cascading style sheets (CSS) - the industry standard for page design, font sizing, and style | 15% | | | 8% | 4% | 5% |
| Missing files | Assessed the absence to referred files on the page, resulting in failed page construction | | | | 8% | 3% | 2% |
| Open Graph | Open Graph tags are the emerging standard to facilitate sharing of web content on social media | | | 4% | 6% | 4% | 3% |
| Popularity | Calculates the relative Alexa ranking of popularity compared to other websites and whether the ranking is rising or falling; Alexa combines the viewing history of many web browsers using particular browser toolbars | | | 9% | | 6% | 4% |
| Printability | Determines whether a web page is designed to be printed and whether specific CSS stylesheets are designed for printing | | | | 8% | 5% | 3% |
| Readability | Assesses the comprehensibility and quality of site content using the Flesch-Kincaid Reading Ease and Gunning Fog Index scales | 8% | 33% | 2% | | 5% | 7% |
| Redirections | Assesses the ability to move backward and forward across pages using browser buttons | 6% | | | 6% | 1% | 2% |
| Search engine results | Determines how text from a web page appears in Google search results | 4% | 13% | 7% | 4% | 5% | 6% |
| Social interest | Checks the amount of social interest that individual pages within the website have. Heavily reliant on Facebook Likes and Twitter for its calculation. | | | 11% | | 5% | 5% |

| | | | | | | | |
|---|---|---|---|---|---|---|---|
| Speed | Assesses the use of files in the website—specifically, how large the files are, how long they take to download, and whether any are missing | 6% | | 3% | 15% | 10% | 7% |
| Spelling | Determines whether the words on a page are spelled correctly | | 20% | | | | 2% |
| Stylesheets | Determines whether and how effectively CSS is used throughout the site, such as avoiding embedded stylesheets and tables for website positioning | 8% | | 1% | 6% | 5% | 4% |
| Twitter | Determines whether the website has a Twitter account and how often the account is referred to | | | 7% | | 3% | 3% |
| URL format | Assesses the use of natural language to improve the readability of the address for each page | 7% | | 7% | 4% | 4% | 5% |
| W3C compliance | Assesses whether the website implements best practices and meets specifications for site development according to the W3C, a consortium and standards body of web technologies | 8% | | | 8% | 5% | 4% |
