# Supplementary material for: Hospital Website Rankings in the United States: Expanding Benchmarks and Standards for Effective Consumer Engagement
Source: J Med Internet Res. 2014 Feb 25;16(2):e64. doi: 10.2196/jmir.3054 (PMC3961706; doi:10.2196/jmir.3054)
Supplement: Supplementary file 2 [file jmir_v16i2e64_app2.pdf]

Multimedia Appendix 2
Ranking of the Top 100 Websites for each dimension and an average ranking across dimensions

	Accessibility		Content		Marketing		Technology		Usability		Overall	
	Score	Rank	Score	Rank	Score	Rank	Score	Rank	Score	Rank	Averaged Rank	Overall Rank
jaxhealth.com	8.5	15	7.5	61	7.3	52	8.3	14	7.9	6	29.6	1
avera.org	8.1	44	7.5	61	7.5	30	8.2	22	7.8	13	34	2
mercycare.org	8.7	6	7.5	61	7.3	52	7.9	51	7.9	6	35.2	3
nationaljewish.org	8.2	33	7.5	61	7.8	7	7.8	70	7.9	6	35.4	4
nmh.org	7.8	91	7.7	19	8.1	2	7.8	70	8	1	36.6	5
pellahealth.org	8.7	6	7.4	107	7.2	72	8.5	5	7.9	6	39.2	6
uabmedicine.org	8	58	7.5	61	7.6	19	7.9	51	7.8	13	40.4	7
nebraskamed.com	8.2	33	7.4	107	7.5	30	8.1	29	7.8	13	42.4	8
slhn.org	8.6	9	7.4	107	7	107	8.3	14	7.9	6	48.6	9
stlukescr.org	7.8	91	7.6	40	7.3	52	7.8	70	7.6	26	55.8	10
tricitymed.org	8.2	33	7.5	61	6.9	140	8.1	29	7.7	18	56.2	11
midmichigan.org	8.6	9	7.2	256	7.8	7	8.3	14	8	1	57.4	12
baptistonline.org	8.9	2	7.2	256	7.4	41	8.4	9	8	1	61.8	13
crozer.org	8.8	4	7.3	167	6.9	140	8.7	1	7.9	6	63.6	14
jhsmh.org	8	58	7.3	167	7.1	82	8	36	7.5	38	76.2	15
neabaptist.com	9	1	7.3	167	6.7	205	8.5	5	7.7	18	79.2	16
stjohnprovidence.org	7.7	126	7.4	107	7.1	82	7.9	51	7.5	38	80.8	17
stanthonysmedcenter.com	8.5	15	7.2	256	6.9	140	8.5	5	7.6	26	88.4	18
sutterhealth.org	7.2	381	7.5	61	7.8	7	7.8	70	7.8	13	106.4	19
southernregional.org	8.1	44	7.2	256	6.8	173	8	36	7.6	26	107	20
nkch.org	8.2	33	7.2	256	6.9	140	7.8	70	7.5	38	107.4	21
ynhh.org	7.6	163	7.2	256	7.6	19	7.7	99	7.6	26	112.6	22
augustahealth.com	8.7	6	7.3	167	6.3	372	8.5	5	7.6	26	115.2	23
sthelenahospitals.org	8.3	24	7.4	107	6.3	372	8.1	29	7.3	65	119.4	24

cheshire-med.com	7.7	126	7.3	167	6.8	173	7.8	70	7.3	65	120.2	25
mymethodist.net	8.4	19	7.1	398	6.8	173	8.1	29	7.7	18	127.4	26
paloscommunityhospital.org	8.1	44	7.2	256	6.8	173	7.6	118	7.4	52	128.6	27
mills-peninsula.org	7.1	442	8	7	7.5	30	7.5	151	7.7	18	129.6	28
cpmc.org	7.1	442	7.5	61	7.5	30	7.7	99	7.7	18	130	29
amh.org	8.9	2	7	538	7	107	8.7	1	7.9	6	130.8	30
whitememorial.com	8.3	24	7.1	398	6.8	173	8.2	22	7.5	38	131	31
thedacare.org	7.3	307	7.5	61	7	107	7.5	151	7.5	38	132.8	32
adventisthealthcare.com	8	58	7.2	256	7.2	72	7.2	241	7.4	52	135.8	33
ketteringhealth.org	8	58	7.2	256	6.4	329	8.3	14	7.6	26	136.6	34
iowahealth.org	7.5	213	7.7	19	7.6	19	6.7	355	7.1	115	144.2	35
lexmed.com	8.1	44	7.2	256	6.9	140	7.3	205	7.2	92	147.4	36
nyhq.org	7.4	250	7.7	19	6.6	249	7.5	151	7.1	115	156.8	37
bethesdaweb.com	7.6	163	7.2	256	6.7	205	7.7	99	7.3	65	157.6	38
integrisok.com	7.9	72	7.4	107	7.1	82	6.5	413	7.1	115	157.8	39
porthuronhospital.org	7.6	163	7.1	398	6.9	140	8	36	7.4	52	157.8	39
missionrmc.org	7.4	250	7.2	256	6.8	173	7.9	51	7.3	65	159	41
mercy.net	8	58	6.9	684	7.6	19	7.9	51	7.7	18	166	42
harthosp.org	7.9	72	7.2	256	7.5	30	6.5	413	7.3	65	167.2	43
genesishealth.com	8	58	7.1	398	6.5	293	7.9	51	7.5	38	167.6	44
bozemandeaconess.org	7.8	91	7.5	61	6.7	205	6.8	331	6.9	178	173.2	45
cancercenter.com	7.1	442	7.3	167	7.7	14	7.3	205	7.5	38	173.2	45
memorialhealth.us	7.6	163	7.3	167	7.6	19	6.5	413	7.1	115	175.4	47
comhs.org	8.1	44	7	538	6.5	293	8.4	9	7.6	26	182	48
sharp.com	7.3	307	7.5	61	7.5	30	6.4	438	7.2	92	185.6	49
allinahealth.org	7.6	163	7.4	107	6.6	249	7.1	266	7	146	186.2	50
multicare.org	6.7	709	7.9	10	6.9	140	8	36	7.5	38	186.6	51
iuhealth.org	8.1	44	6.9	684	7.8	7	7.4	173	7.5	38	189.2	52
crmchealth.org	7.6	163	7.2	256	6.3	372	7.8	70	7.2	92	190.6	53
bonsecours.com	7.5	213	7	538	7	107	7.8	70	7.6	26	190.8	54

rushcopley.com	8.8	4	6.8	876	7.2	72	8.7	1	7.8	13	193.2	55
pamf.org	6.9	570	8.2	4	8	4	6.7	355	7.5	38	194.2	56
mayoclinic.org	8	58	6.8	876	8.5	1	7.9	51	8	1	197.4	57
asante.org	7.9	72	7.1	398	6.3	372	7.8	70	7.2	92	200.8	58
urmc.rochester.edu	7.6	163	7	538	7.6	19	7.1	266	7.6	26	202.4	59
sonoramedicalcenter.org	7.4	250	7.5	61	6	544	7.9	51	7.1	115	204.2	60
altabates.org	7.1	442	7.7	19	6.4	329	7.6	118	7.1	115	204.6	61
svch.net	7.6	163	7.3	167	6.5	293	7.1	266	7	146	207	62
ucsfhealth.org	8.2	33	6.8	876	7.5	30	7.8	70	7.6	26	207	62
northshorelij.com	7	506	7.5	61	7.5	30	6.6	378	7.3	65	208	64
adena.org	7.8	91	7.1	398	6.5	293	7.4	173	7.2	92	209.4	65
stemc.org	7.8	91	7.3	167	6.2	419	7.3	205	6.9	178	212	66
emoryhealthcare.org	7.6	163	6.8	876	8.1	2	8.2	22	8	1	212.8	67
scripps.org	6.8	631	8.3	3	7.9	5	6.6	378	7.3	65	216.4	68
montefiore.org	6.9	570	7.2	256	7.2	72	7.5	151	7.4	52	220.2	69
southernoceanmedicalcenter.com	7.6	163	7.4	107	5.9	616	7.8	70	7	146	220.4	70
chomp.org	8.6	9	7	538	6.3	372	7.6	118	7.3	65	220.4	70
meriter.com	7.7	126	6.8	876	7.7	14	7.8	70	7.6	26	222.4	72
westhoustonmedical.com	7.5	213	7.4	107	6.4	329	7	287	6.9	178	222.8	73
rrmc.org	8.4	19	7.2	256	5.7	755	8.2	22	7.3	65	223.4	74
essentiahealth.org	7	506	8.6	1	7.1	82	6.4	438	7.2	92	223.8	75
genesishcs.org	8.3	24	6.8	876	6.9	140	8.1	29	7.4	52	224.2	76
guthrie.org	8.3	24	7.2	256	6.3	372	6.6	378	7.2	92	224.4	77
beloithealthsystem.org	7.7	126	7.1	398	6.2	419	7.7	99	7.2	92	226.8	78
tuomey.com	7.2	381	7.4	107	6.2	419	7.7	99	7	146	230.4	79
miamivalleyhospital.org	7.8	91	7	538	6.8	173	7.4	173	6.9	178	230.6	80
carilionclinic.org	7.4	250	6.9	684	7.2	72	7.7	99	7.4	52	231.4	81
parmahospital.org	7.4	250	7	538	6.7	205	7.6	118	7.3	65	235.2	82
winonahealth.org	8.1	44	6.8	876	7.1	82	7.6	118	7.3	65	237	83
greenhosp.org	7.2	381	7.1	398	6.7	205	7.6	118	7.2	92	238.8	84

brighamandwomens.org	7.3	307	7.7	19	7.1	82	5.9	676	7.1	115	239.8	85
sibley.org	7.8	91	7.1	398	6.8	173	6.6	378	6.9	178	243.6	86
robinsonmemorial.org	7.6	163	7.6	40	5.9	616	7.4	173	6.8	232	244.8	87
ochsner.org	7.2	381	7.1	398	7.3	52	6.8	331	7.3	65	245.4	88
emhc.org	7.3	307	7	538	6.6	249	7.8	70	7.3	65	245.8	89
sutterlakeside.org	6.9	570	7.5	61	6.3	372	7.6	118	7.1	115	247.2	90
danburyhospital.org	6.7	709	7.4	107	6.6	249	7.6	118	7.3	65	249.6	91
regionshospital.com	8.3	24	6.7	1082	7.7	14	7.6	118	7.5	38	255.2	92
riversidehealthcare.org	7.6	163	7.1	398	7.4	41	6.2	530	7	146	255.6	93
marylanning.org	7.3	307	7.4	107	5.8	681	7.8	70	7.1	115	256	94
wpahs.org	8	58	6.7	1082	7.8	7	7.8	70	7.3	65	256.4	95
northshore.org	7.5	213	6.9	684	7.2	72	7.1	266	7.4	52	257.4	96
holy-cross.com	7	506	8	7	7.1	82	6.1	579	7.1	115	257.8	97
medicalcityhospital.com	7.2	381	7.5	61	6.9	140	6.2	530	6.9	178	258	98
evergreenhealth.com	7.3	307	7.1	398	6.7	205	7.1	266	7.1	115	258.2	99
baptist-health.org	7	506	7.2	256	7	107	6.9	309	7.1	115	258.6	100
